# Endothelial prostacyclin protects the kidney from ischemia-reperfusion injury

**DOI:** 10.1007/s00424-018-2229-6

**Published:** 2018-11-09

**Authors:** Yingxue Cao, Yi Guan, Yun-Yu Xu, Chuan-Ming Hao

**Affiliations:** Division of Nephrology, Huashan Hospital, Fudan University, 12 Wulumuqi Road (middle), Shanghai, 200040 China

**Keywords:** Prostacyclin, Ischemia-reperfusion injury, IP receptor, Renal blood flow

## Abstract

Prostacyclin, or PGI_2_, is a product of PGI synthase (PGIS), down-stream of cyclooxygenase pathway. PGI_2_ has been demonstrated to play an important role in maintaining renal blood flow. Non-steroidal anti-inflammatory drugs (NSAIDs) that inhibit cyclooxygenase are reported to increase the susceptibility of patients to acute kidney injury (AKI). This study explores the role of endothelium-derived prostacyclin in ischemia-reperfusion injury (I/RI). The renal PGIS expression and PGI_2_ production markedly increased following I/RI. Loss of one allele of PGIS gene or selective endothelial PGIS deletion (TEK-CRE PGIS^fl/fl^ mice) caused more severe renal damage following I/RI than control mice. Iloprost, a PGI_2_ analog, administered 30 min before the I/R surgery, markedly attenuated the renal damage in both control mice and TEK-CRE PGIS^fl/fl^ mice. Renal p-PKA expression significantly increased after I/RI in wild-type mice but not in the PGIS deletion mice, consistent with IP receptor mediating the protective effect. Further studies showed that PGIS deficiency was associated with reduced fluorescence microsphere accumulation in the kidney following I/R. Folic acid also induced marked kidney injury; however, endothelial PGIS deletion did not worsen kidney injury compared with wild-type mice. These studies indicate that PGIS-derived PGI_2_ can protect the kidney from acute injury caused by ischemia and reperfusion and PGIS/PGI_2_ is a potential intervention target for AKI.

## Introduction

Acute kidney injury (AKI) is a global public concern that is associated with high morbidity, mortality, and healthcare costs, and is increasingly prevalent in both developing and developed countries [24, 27, 42]. Ischemia-reperfusion injury (I/RI) is the most common cause of AKI [2, 43]. Although extensive studies have been performed over the past decades, the incidence of AKI remains high, and therapies are limited. Furthermore, renal I/RI is almost unavoidable in renal transplantation [25], which may contribute significantly to delayed graft function (DGF). Additionally, up to 30% of the patients receiving cardiac surgery may experience acute kidney injury [36]. Other than dialysis, no pharmacological treatments reliably improve survival, limit injury, or accelerate recovery [47].

Prostacyclin, or PGI_2_, is a member of the prostaglandin family. PGI_2_ is one of the end metabolites of arachidonic acid that is produced via cyclooxygenase (COX) and PGI synthase (PGIS) [5, 32, 37]. PGI_2_ has a half-life of 30 s and is metabolically degraded to 6-Keto PGF_1α_. The short half-life limits its effect to the site of synthesis [19, 29]. As an endogenous vasodilator and inhibitor of leukocyte adhesion and platelet aggregation, PGI_2_ acts mainly on the membrane-bound IP receptor [9] and signals by increasing the intracellular cyclic AMP (cAMP) level [4, 9, 19, 31], which leads to the activation of protein kinase A (PKA) and further phosphorylation of related proteins that exert a variety of biologic functions [5, 12, 20, 21]. PGI_2_ can also bind to endogenous peroxisome proliferator-activated receptors (PPARs) and regulate the transcription of target genes [6, 40]. NSAIDs that inhibit COX and thus prostaglandin production have been reported to increase patient’s susceptibility to AKI [3, 10, 34, 35]. It has been reported that deletion of PGIS is associated with ischemic renal disorders, including nephrosclerosis and renal infarction [45]. Furthermore, in a study of rats, PGI_2_ protected the kidney from post-ischemic acute injury, although the mechanism is incompletely characterized [15]. These findings suggest that PGI_2_ plays an important role in maintaining renal blood flow and protecting the kidney from acute injury.

Therefore, the present study was designed to test the hypothesis that PGIS/PGI_2_ pathway is responsible for maintaining renal homeostasis during ischemic reperfusion and protecting the kidney from AKI. This study may shed light on the mechanism underlying AKI and the therapeutic potential of the PGI_2_ pathway during I/RI.

## Materials and methods

### Animals

All animal studies were approved by the Institutional Animal Care and Use Committee of Fudan University. Wild-type (WT) C57BL/6 mice were purchased from Biomodel Organism (Shanghai, China). Mice were housed at a constant temperature and humidity room in the animal facility of Fudan University Medical Animal Center with a 12:12-h light-dark cycle and allowed free access to standard rodent chow and water.

**Fig. 1:**
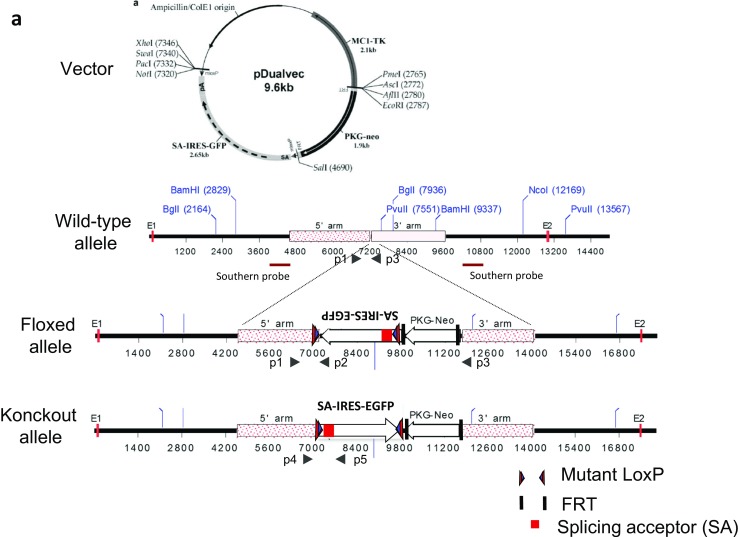

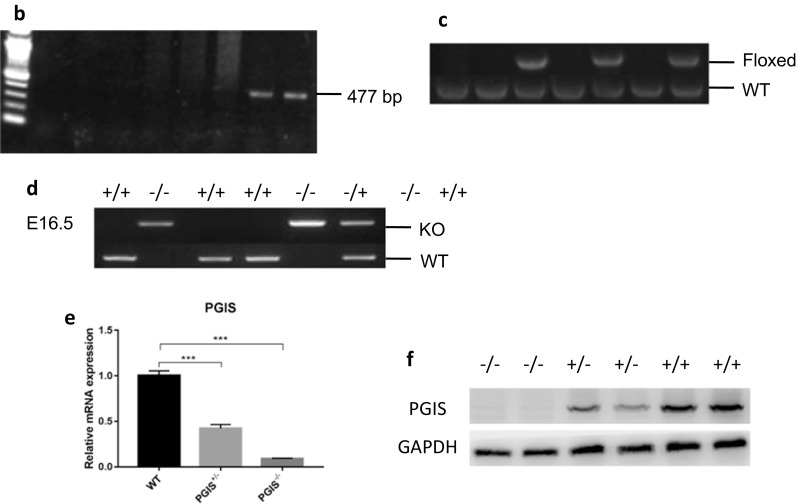
Generation and confirmation of PGIS-floxed mice. **a** Targeting strategy. The targeting construct has a 4-kb 5′ homogenous arm, a reversed sequence including an SA and an IRES-EGFP reporter flanked by two reverse loxP sequence, a PGK-Neo selection cassette flanked by FRT, and a 2-kb 3′ homogenous arm. **b** Southern blot showed two targeted ES cell clones that give expected hybridization patterns. The 3′ probe indicated in **a** was used to confirm that the correct target was obtained. **c** Identification of PGIS-floxed mice by PCR. **d** Determination of conventional knockout E16.5 embryos’ genotypes by PCR. PGIS production in the body of E16.5 embryos was analyzed by real-time PCR (**e**) and Western blot (**f**). A total of 11 mice were included (*n* = 4 in the WT group; *n* = 4 in the PGIS^+/−^ group, and *n* = 3 in the PGIS^−/−^ group). The data are expressed as the mean ± SEM and were analyzed using one-way ANOVA among multiple groups followed by Scheffe post hoc testing. ***P < 0.001. WT, wild-type; KO, knockout

The Cre/loxP and Flp/FRT recombination system was used to generate PGIS-floxed mouse line. The mouse genomic PGIS gene sequence was obtained from the UCSC Genome Browser. The targeting vector included a 4-kb 5′ homogenous arm, a reversed sequence including a splicing acceptor (SA) and an IRES (internal ribosome entrance site) tagged with EGFP (enhanced green fluorescence protein) and flanked by two reverse loxP sequence, a PGK-Neo selection cassette flanked by FRT, and a 2 kb 3′ homogenous arm. The IRES-EGFP cDNA in the targeting construct allows for detecting where the floxed PGIS is expressed in mice carrying the transgene. The target vector was transfected into embryonic stem cells following established protocols [39]. Neomycin-resistant embryonic cell clones were screened by Southern blot, and two correctly targeted clones were identified (Fig. 1b). Two target clones (1C11 and 3D8) were picked for blastocyst injection. Germline transmission of the transgene was obtained from the 1C11 line. Genotyping was achieved through PCR of tail DNA with primers: P1 5′- CTGTCCCTATCTAAACCTCACC-3′, P2 5′-TAGAGCGGCCATCATAACT-3′, P3 5′-GCCAAGCATTCGTAAAGCCC-3′. PCR was performed under the following conditions: denaturation at 95 °C for 3 min; 35 cycles of 94 °C for 30 s, 58 °C for 1 min, and 72 °C for 1 min; and 72 °C for 5 min. Primers P1 and P3 amplify an 857-bp wild-type allele fragment, and primers P1 and P2 amplify a 577-bp floxed allele fragment.

Mice with PGIS gene deletion were obtained by crossing the floxed PGIS mouse with a universal Cre mouse (the EIIA-Cre mouse on the B6 background). Most of the homozygous PGIS knockout mice (PGIS^−/−^) experienced embryonic lethality. The heterozygous (PGIS^+/−^) mice were further bred with WT C57BL/6 mice to expand the heterozygous PGIS knockout mice (PGIS^+/−^) and WT littermates (PGIS^+/+^). Genotyping was achieved with primers P4 5′-ACAGGGTTTCATCCTCAATT-3′, P5 5′- GTCCTTATCATCGTCGTCTTT-3′, which amplified a 900-bp knockout allele fragment.

The endothelial-specific PGIS-deficient mouse line was generated by mating the PGIS^fl/fl^ mice with the endothelial Cre TEK-CRE mouse line. Mice with both floxed PGIS alleles and the TEK-CRE transgene were generated (TEK-CRE PGIS^fl/fl^). TEK-CRE PGIS^fl/fl^ mice were then backcrossed to the PGIS^fl/fl^ mice to generate both deletable and nondeletable littermates.

A smooth muscle cell-specific PGIS-deficient mouse line was generated by mating the PGIS^fl/fl^ mice with the SMMHC-iCRE mouse line. Mice with both floxed PGIS alleles and the SMMHC-iCRE transgene were generated (SMMHC-iCRE PGIS^fl/fl^). SMMHC-iCRE PGIS^fl/fl^ mice were then backcrossed to the PGIS^fl/fl^ mice to generate both deletable and nondeletable littermates.

### Experimental model of AKI and drug treatment

Warm ischemia-reperfusion surgery was performed on 8- to 10-week-old male C57BL/6 mice. Mice were subjected to flank incisions. Ischemia was induced after right-sided uninephrectomy by clipping the pedicles of the remaining left kidney for 25 min. Reperfusion was confirmed visually. The color of the kidney turned from dark purple to pink. Sham surgery was performed in a similar manner, except for clamping of the renal vessels. Iloprost (50 mg/kg body wt) or an equivalent amount of PBS was administered 30 min before the surgery by intraperitoneal injection. In the nephrotoxic model, folic acid was dissolved in 0.2 ml of 0.3 mM sodium bicarbonate (NaHCO_3_) and administered intraperitoneally at a concentration of 250 mg/kg/wt. To collect blood and tissue samples, mice were anesthetized with chloral hydrate (400 mg/kg, intraperitoneal injection).

### Renal blood flow evaluation

Red fluorescent 15 μm FluoSpheres (Molecular Probes) were used. Fifty microliters of the microsphere mixture was injected into the left atrium of live mice after IR surgery.

#### In vivo imaging system

Mice were anesthetized with 5% chloral hydrate. The abdomen was opened to fully expose the kidney. In vivo images were acquired with using an In Vivo Imaging System (NightOWL LB 983, Berthold Technologies, Germany).

#### Quantitation of fluorescence

The animals were sacrificed, and the kidneys were removed for fluorescence quantification after digestion and filtration, according to the manufacturer’s instructions. The intensity of the fluorescence signal was determined with an EnSpire luminescence spectrophotometer (Perkin-Elmer, Beaconsfield, UK) equipped with a 96-well microplate reader. The fluorescence intensity was measured at the optimal excitation-emission wavelengths of red dye (570 and 598 nm).

### Prostaglandin production assessment

The production of PGI_2_ was assessed by measuring 6-Keto PGF_1α_ using an ELISA Kit (No. 515211; Cayman Chemical Company, Ann Arbor, MI) according to the manufacturer’s instructions. Thromboxane A_2_ was assessed by measuring thromboxane B_2_ using an ELISA Kit (No. 501020; Cayman Chemical). PGE_2_ was also measured with an ELISA Kit (514,010; Cayman Chemical).

### Measurement of blood pressure

Mice were restrained in a mouse pocket and maintained at 37 °C. Each mouse had their blood pressure continuously measured 10 times per day for a week by the tail-cuff method with a noninvasive automatic blood pressure analyzer (BP-2000 Blood Pressure Analysis System, Visitech Systems, USA), and the mean results were used.

### Renal function evaluation

Renal function was evaluated by blood urea nitrogen (BUN). BUN was measured using a UREA KIT (liquid; UV-GLDH method; Shanghai Kehua Bioengineering Co., Ltd.) according to the manufacturer’s instructions.

### Kidney histology

Kidney samples were fixed in 4% paraformaldehyde, and paraffin-embedded, 2-μm sections were stained with periodic acid-Schiff. Over six low-power fields of each section were reviewed and scored for tubular injury in a single-blind fashion using a modified one to four scoring system, as described previously [1].

### Immunofluorescence

Paraformaldehyde-fixed, paraffin-embedded, 2-μm-thick sections were deparaffinized in xylene and rehydrated in a graded alcohol series. To retrieve the antigen, sections were heated in citrate buffer (pH 6.0) for 15 min at 120 °C by means of an autoclave. Sections were then blocked with 5% BSA and incubated overnight at 4 °C with the following primary antibodies: mouse anti-PGIS (1:100; Cayman Chemical) and rat anti-CD34 (1:200; Abcam, Cambridge, UK). For fluorescent visualization of the bound primary antibodies, sections were further incubated with the appropriate Cy3-conjugated or FITC-conjugated secondary antibodies (1:200; Jackson ImmunoResearch Laboratories) for 1 h at room temperature.

### Electron microscopy

Kidney tissues were fixed in 2.5% glutaraldehyde and 2.5% paraformaldehyde in phosphate buffer. After a standard embedding procedure, the peritubular capillaries were examined and photographed under a transmission electron microscope.

### TUNEL assay

Apoptotic cells were detected by TUNEL assay (G3250; Promega, Fitchburg, WI) according to the manufacturer’s instruction, and the number of TUNEL-positive cells in five sections per kidney was quantified. DAPI was used as a counterstain.

### Real-time PCR

Total RNA was extracted from the kidney tissue using TRIzol Reagent (Invitrogen, Carlsbad, CA), and 1 μg RNA was reverse transcribed using oligo dT as the primer according to the manufacturer’s instructions (RR037; Takara Bio, Dalian, China). Real-time PCR was performed using the SYBR Green Premix Kit (RR820; Takara Bio, Dalian, China). The expression level of genes of interest was calculated with a comparative method (2^−DDCT^) using β-actin as an internal control. The following primers were obtained from the primer bank: PGIS, forward 5′-CTGGTTGGGGTATGCCTTGG and reverse 5′- TCATCACTGGGGCTGTAATGT; β-actin, forward 5′- GGCTGTATTCCCCTCCATCG and reverse 5′- CCAGTTGGTAACAATGCCATGT.

### Western blotting

Protein was extracted from frozen kidney tissues using RIPA lysis buffer (P0013B; Beyotime, Nantong, China) with a phosphatase inhibitor tablet (Roche Diagnostics GmbH, Mannheim, Germany), a proteinase inhibitor cocktail tablet (Roche), and 100 mM phenylmethanesulfonyl fluoride (ST506; Beyotime), and the ratio of the listed chemicals to the RIPA lysis buffer was 1:100. Protein concentration was determined using the Bradford protein assay (P0011; Beyotime). After the proteins were separated via SDS-PAGE, they were transferred electrophoretically to PVDF membranes (Millipore, Billerica, MA) and blocked with 5% nonfat milk or BSA. Then, immunoblotting was performed using specific antibodies overnight at 4 °C: rabbit anti-PGIS (1:500; Abcam), anti-PKA (1:1000; CST, Danvers, MA), anti-P-PKA (1:1000; CST), anti-β-actin (1:5000; CST), and anti-α-tubulin (1:8000; Abcam). Then, the membranes were incubated with horseradish peroxidase-conjugated anti-rabbit or anti-mouse Ig for 1 h at room temperature. Antibody labeling on the Western blots was visualized using a chemiluminescence reagent (WBKLS0100; Millipore) with GE ImageQuant LAS 4000. Densitometry analysis was performed using ImageJ software.

### Statistical analyses

All values are expressed as the mean ± SEM. Statistical comparisons were made by Student’s unpaired *t* test or one-way ANOVA with post hoc analysis. GraphPad Prism 7 and SPSS 24 software were used for all calculations. A two-sided *P* value of less than 0.05 was considered significant.

## Results

### Generation of PGIS-floxed mice

To examine the role of PGIS and PGI_2_ during kidney I/RI, we developed a PGIS-floxed mouse line. The SA-IRES-EGFP fragment and a PGK-Neo selection cassette were inserted between PGIS exon 1 and exon 2 to inactivate the PGIS gene (Fig. 1a). Neo-resistant embryonic cell clones were screened by Southern blot (Fig. 1b). The mouse genotype was identified by PCR (Fig. 1c). To confirm whether the PGIS-floxed mice were successfully generated, we mated the PGIS^fl/+^ mouse with the EIIA-cre mouse to produce conventional PGIS knockouts. The homozygous PGIS knockout mice were embryonic lethal after E16.5 days. We analyzed the expression of PGIS mRNA and PGIS protein in the E16.5 embryos by real-time PCR and Western blot, respectively (Fig. 1d–f). The expression of PGIS mRNA was significantly reduced in heterozygous embryos and was absent in homozygous embryos. Likewise, the PGIS protein was undetectable in homozygous embryos, indicating that the PGIS-floxed mice were successfully established.

### PGIS deficiency aggravates I/R-induced AKI

**Fig. 2:**
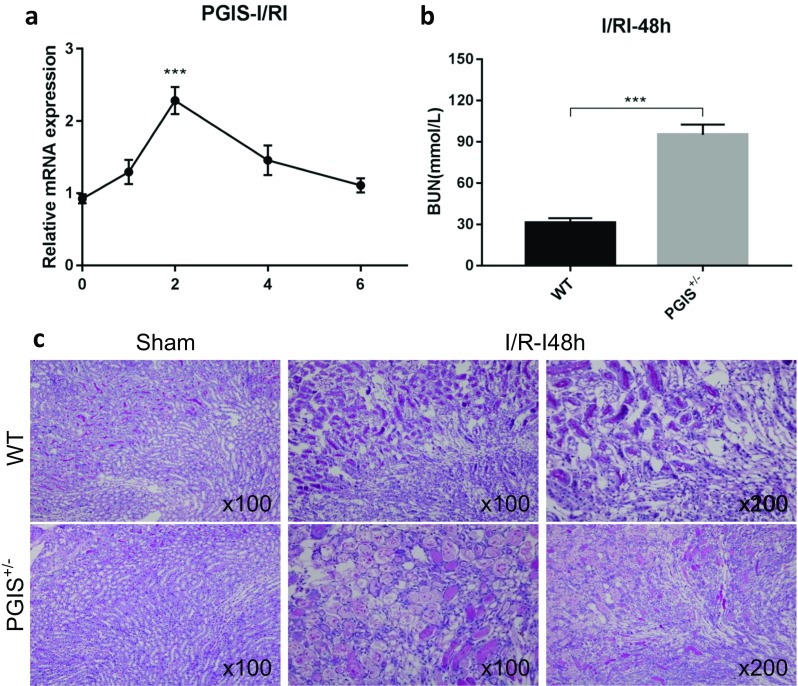
The PGIS-deficient kidney is susceptible to AKI. WT and PGIS^+/−^ mice were subjected to right uninephrectomy and sham surgery or 25 min of left renal ischemia. **a** The relative mRNA expression of PGIS was measured at the indicated times. A total of 20 mice were included (*n* = 4 per time point). **b** BUN concentrations were measured at 48 h after ischemia. A total of 10 mice were included (*n* = 4 in the WT group and *n* = 6 in the PGIS^+/−^ group). **c** Renal tubular injury is indicated by representative periodic acid-Schiff staining of kidney sections at both low and high magnification. The data are expressed as the mean ± SEM and were analyzed by Student’s unpaired *t* test between two groups and one-way ANOVA among multiple groups followed by Dunnett’s post hoc testing. ***P < 0.001; WT, wild type; CON, control

The PGIS expression significantly increased at 48 h after I/R (Fig. 2a). To determine the role of PGIS in kidney injury, studies were performed in the PGIS heterozygous mice. Deletion of one allele of the PGIS gene significantly aggravated renal damage compared with wild-type littermates at 48 h after ischemia, resulting in a higher BUN levels and more severe tubular injury (Fig. 2b, c).

### Role of endothelial PGIS in AKI

**Fig. 3:**
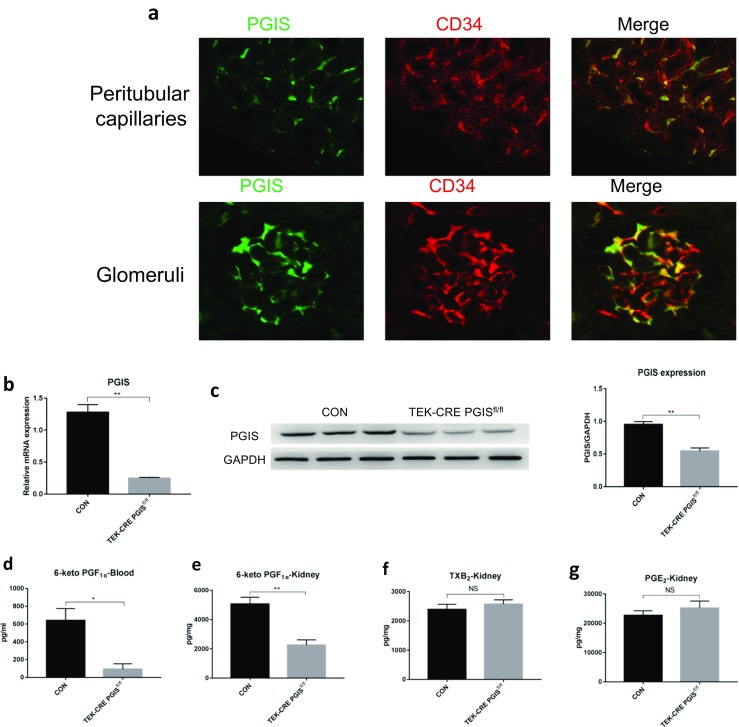
Prostanoid production in endothelial-specific PGIS-deficient mice. **a** Costaining of PGIS with endothelial cell marker CD34 in wild-type kidney sections. **b** Relative mRNA expression level of PGIS in the kidneys of control (CON) and endothelial-specific PGIS-deficient (TEK-CRE PGIS^fl/fl^) mice (*P* = 0.001). **c** Representative Western blot image of PGIS protein expression in the kidney. GAPDH was used as a loading control. Band density was measured using ImageJ software (*P* = 0.003). **d** 6-Keto PGF_1α_ level in the plasma (*P* = 0.020). 6-Keto PGF_1α_ (**e**) (*P* = 0.001), TXB_2_ (**f**), and PGE_2_ (**g**) levels in the kidney. The experiments were repeated twice. A total of 12 mice were included (*n* = 6 per group). The data are expressed as the mean ± SEM and were analyzed by Student’s unpaired *t* test. *P < 0.05; **P < 0.01; NS, not significant

By immunofluorescence, PGIS was found colocalized with CD34, an endothelial cell marker. PGIS was expressed in the cytoplasm of endothelial cells both in the peritubular capillaries and the glomeruli (Fig. 3a). To determine the specific role of endothelial PGIS and PGI_2_ during renal I/RI, we generated a mouse that was deficient in PGIS specifically within the endothelium by mating the floxed PGIS mouse with an endothelial Cre mouse, the TEK-CRE mouse. PGIS expression was significantly decreased both at the mRNA and protein levels in endothelial-specific PGIS-deficient mice (TEK-CRE PGIS^fl/fl^) compared with control mice (Fig. 3b, c). The production of PGI_2_ was also significantly reduced both in the blood and kidney compared with the control group, as assessed by 6-Keto PGF_1α_, a stable metabolite of PGI_2_ (Fig. 3d, e). We also measured TXB_2_, a stable metabolite of TXA_2_, and PGE_2_ in kidneys (Fig. 3f, g). Deleting the PGIS gene in endothelial cells did not influence the expression of other genes in the prostaglandin family.

**Fig. 4:**
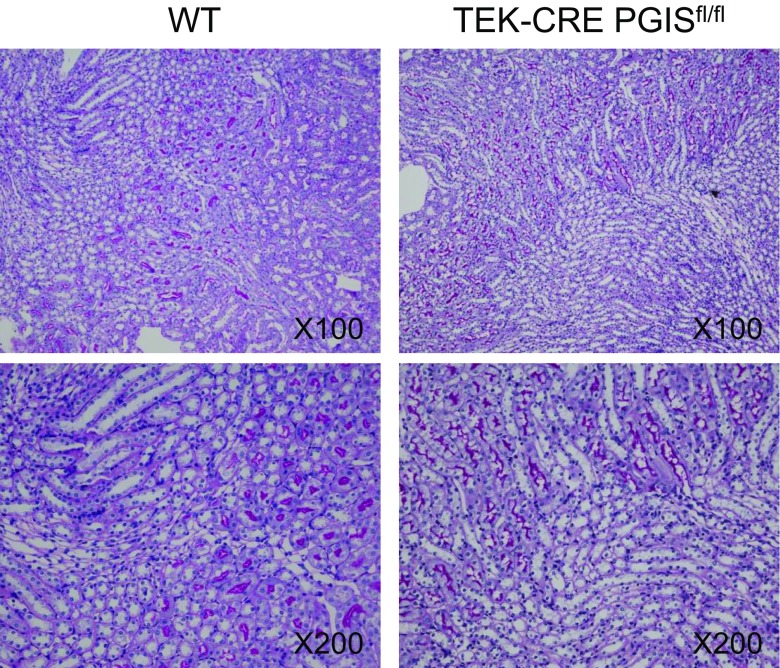
Morphology of the kidneys of wild-type and TEK-CRE PGIS^fl/fl^ mice (periodic acid-Schiff stain)

**Fig. 5:**
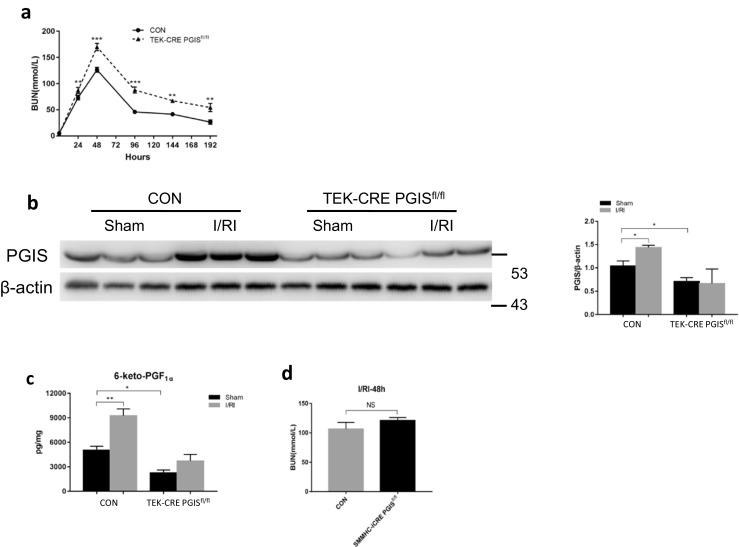
Endothelial PGIS protects the kidney from AKI. The control mice, TEK-CRE PGIS^fl/fl^ mice, and SMMHC-iCRE PGIS^fl/fl^ mice were subjected to either renal ischemia or a sham operation. **a** BUN concentrations at the indicated times. A total of eight mice were included (*n* = 4 per group). **b** Representative Western blot images of PGIS protein expression in the kidney (in the control group, *P* = 0.033 Sham vs. I/RI). β-Actin was used as a loading control. Band density was measured using ImageJ software. **c** The production of 6-Keto PGF_1α_ in the kidneys (in the control group, *P* = 0.001 Sham vs. I/RI). For **b**, **c**, 24 mice were included (CON mice: *n* = 7 in the Sham group, *n* = 6 in the I/RI group; TEK-CRE PGIS^fl/fl^ mice: *n* = 5 in the Sham group, *n* = 6 in the I/RI group). **d** BUN concentrations were measured at 48 h after ischemia. A total of eight mice were included (*n* = 4 per group). The data are expressed as the mean ± SEM and were analyzed by Student’s unpaired *t* test between two groups and one-way ANOVA among multiple groups followed by Scheffe post hoc testing. For **a**, the data were analyzed by Student’s unpaired *t* test followed by Bonferroni correction (a two-sided *P* value less than 0.01 was considered significant). *P < 0.05; **P < 0.01; ***P < 0.001; NS, not significant

No developmental abnormalities were observed in the kidneys of TEK-CRE PGIS^fl/fl^ mice (Fig. 4). The blood pressure and heart rates were comparable between wild-type mice and TEK-CRE PGIS^fl/fl^ mice (Table 1). Following ischemia and reperfusion, the TEK-CRE PGIS^fl/fl^ mice had higher BUN levels than control mice (Fig. 5a). The endothelial-specific PGIS knockout also abolished the increase of PGIS expression and PGI_2_ production after ischemia and reperfusion (Fig. 5b, c). Deletion of the PGIS gene specifically in smooth muscle cells did not worsen the kidney injury compared with wild-type littermates after ischemia and reperfusion (Fig. 5d).

**Table 1:** Systolic blood pressure (BP) and the heart rate of wild-type and TEK-CRE PGIS^fl/fl^ mice. The results are the means ± SEM and were analyzed by Student’s unpaired *t* test. A total of 20 mice were included (*n* = 8 in the wild-type group and *n* = 12 in the TEK-CRE PGIS^fl/fl^ group)

Blood pressure and heart rate of wild-type and TEK-CRE PGIS^fl/fl^ mice		
	Wild type (*n* = 8)	TEK-CRE PGIS^fl/fl^ (*n* = 12)
Systolic BP, mmHg	106 ± 2	99 ± 3
Heart rate, bpm	454 ± 15	426 ± 7

### The PGIS/PGI_2_/IP receptor signaling pathway is activated during I/R-induced AKI

**Fig. 6:**
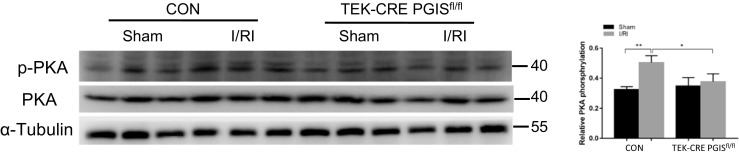
Representative Western blot images of phosphorylated protein kinase A (p-PKA) (in the control group, *P* = 0.006 Sham vs. I/RI). α-Tubulin was used as a loading control. Band density was measured using ImageJ software. A total of 24 mice were included (CON mice: *n* = 7 in the Sham group, *n* = 6 in the I/RI group; TEK-CRE PGIS^fl/fl^ mice: *n* = 5 in the Sham group, *n* = 6 in the I/RI group). The data are expressed as the mean ± SEM and were analyzed by one-way ANOVA among multiple groups followed by Scheffe post hoc testing. *P < 0.05; **P < 0.01

**Fig. 7:**
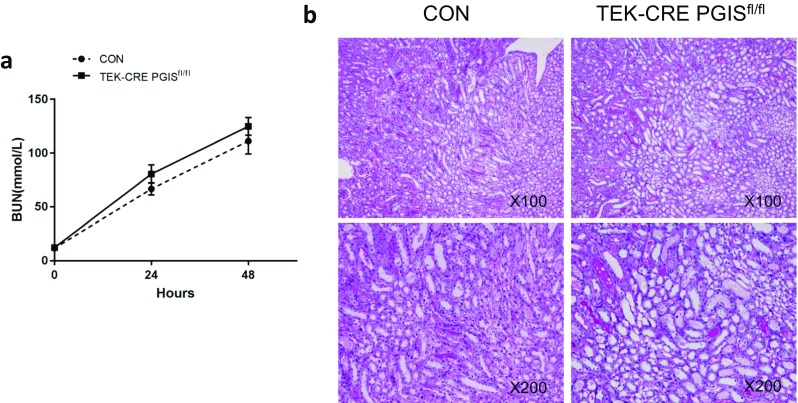
PGI_2_ has no protective effects in a folic acid-induced AKI model. **a** BUN concentrations at the indicated times. **b** Renal tubular injury as indicated by representative periodic acid-Schiff staining of kidney sections at 48 h after folic acid treatment**.** A total of 10 mice were included (*n* = 5 per group). The data are expressed as the mean ± SEM and were analyzed by Student’s unpaired *t* test between two groups

The IP receptor signals mainly through the cAMP/PKA signaling pathway. Following I/R, the level of phosphorylated PKA was significantly increased in the control kidneys but not in the endothelial-specific PGIS knockout kidneys (Fig. 6), consistent with IP/PKA signaling in PGI_2_-associated effect on kidney injury. We then examined the role of PGIS/PGI_2_ in a nephrotoxic AKI induced by folic acid. PGIS deficiency did not worsen renal damage in the folic acid model (Fig. 7).

### Treatment with a PGI_2_ analog protects the kidney from I/R injury

**Fig. 8:**
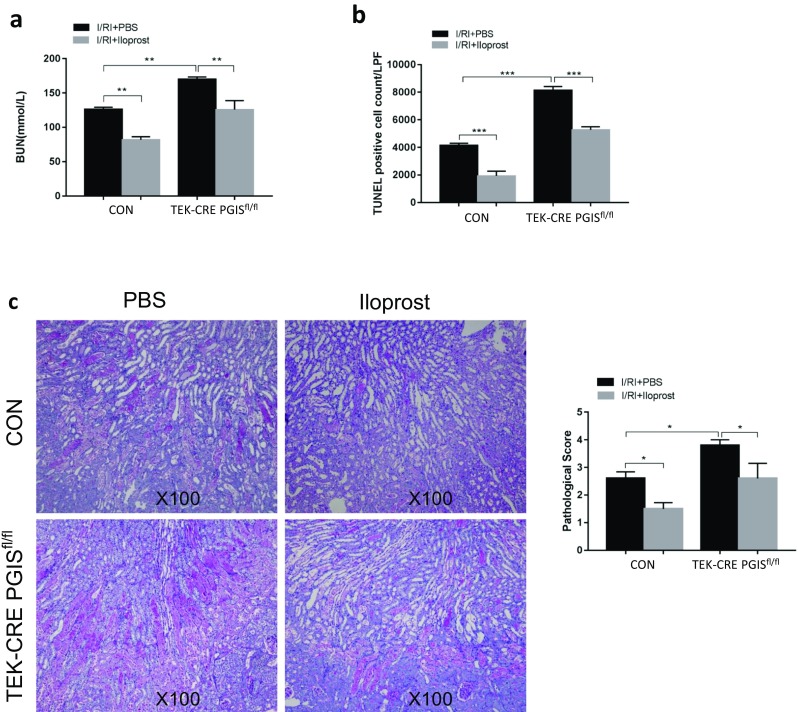
PGI_2_ protects kidneys from I/R-induced AKI. **a** Renal function as evaluated by serum BUN in control and TEK-CRE PGIS^fl/fl^ mice at 48 h after ischemia with or without treatment with the PGI_2_ analog, iloprost (control group: *P* = 0.001 I/RI + PBS vs. I/RI + Iloprost; TEK-CRE PGIS^fl/fl^ group: *P* = 0.002 I/RI + PBS vs. I/RI + Iloprost). Renal tubular injury as indicated by representative periodic acid-Schiff staining of kidney sections and evaluated based on a pathological score (control group: *P* = 0.026 I/RI + PBS vs. I/RI + Iloprost; TEK-CRE PGIS^fl/fl^ group: *P* = 0.019 I/RI + PBS vs. I/RI + Iloprost) (**b**) and TUNEL staining (**c**). A total of 24 mice were included (CON mice: *n* = 6 in the I/RI + PBS group, *n* = 7 in the I/RI + Iloprost group; TEK-CRE PGIS^fl/fl^ mice: *n* = 6 in the I/RI + PBS group, *n* = 5 in the I/RI + Iloprost group). The data are expressed as the mean ± SEM and were analyzed by one-way ANOVA among multiple groups followed by Scheffe post hoc testing. *P < 0.05; **P < 0.01; ***P < 0.001

We further examined whether a PGI_2_ analog, iloprost, can rescue the effect of knocking out PGIS. Iloprost or PBS was administered 30 min before the surgery. BUN measurement, histology analysis, and TUNEL staining were performed at 48 h after ischemia. As shown in Fig. 8, mice treated with iloprost had a significantly lower BUN and improved tubular damage compared with mice treated with PBS both in the control group and the TEK-CRE PGIS^fl/fl^ group, indicating a potential target for AKI treatment.

### Deletion of PGIS gene in endothelial cells reduced renal blood flow after reperfusion

**Fig. 9:**
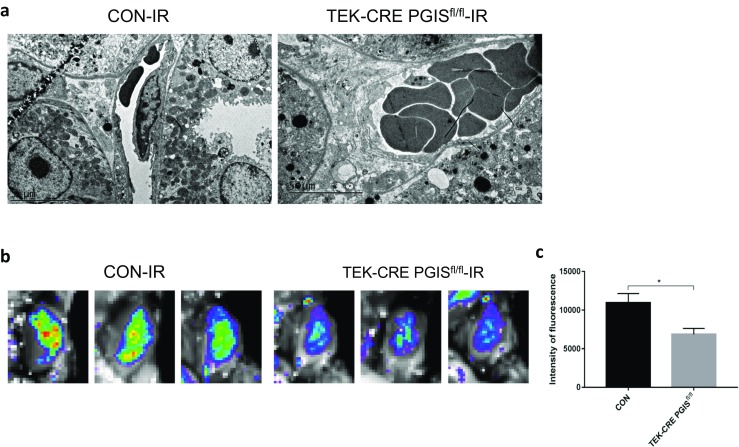
Renal blood flow after reperfusion. **a** Electron microscopy images of the renal peritubular capillaries of the indicated groups are shown. Scale bars, 5 μm. **b** In vivo whole-body imaging of fluorescent microspheres following I/R surgery. **c** Quantification of the fluorescence intensity. A total of 10 mice were included (*n* = 6 in the CON group and *n* = 4 in the TEK-CRE PGIS^fl/fl^ group). The data are expressed as the mean ± SEM and were analyzed by Student’s unpaired *t* test between two groups (*P* = 0.044). *P < 0.05

In the kidneys of TEK-CRE PGIS^fl/fl^ mice, red blood cells were readily observed in the peritubular capillaries at 24 h after ischemia (Fig. 9a). We then examined renal blood flow using a fluorescent microspheres technique. The fluorescent intensity of the kidney was markedly less in the TEK-CRE PGIS^fl/fl^ mice compared with the control mice (Fig. 9b, c).

## Discussion

In the present study, we demonstrated that (1) I/R stimulated the expression of PGIS and PGI_2_; (2) global PGIS deficiency or endothelial-specific PGIS deletion exacerbated I/R-induced injuries; (3) an exogenous PGI_2_ analog protected the kidney from I/R-induced injury; and (4) PGI_2_ deficiency was associated with reduced renal blood flow following reperfusion. These results strongly suggest that endothelium-derived PGI_2_ plays a critical role in protecting the kidney from I/R injury.

It is well documented that prostanoids play an important role in maintaining local homeostasis [18]. PGIS expression markedly increased following I/R injury, and PGIS deficiency significantly exacerbated kidney I/R injury. These results suggest that PGIS/PGI_2_ is involved in the cellular response to I/R stress and protects the kidney from injury. COX inhibitors, which are widely used anti-inflammatory drugs and pain relievers, are associated with AKI. COX-derived products include PGE_2_, PGI_2_, and TXA_2_. The present study suggests that PGI_2_ is an important protective factor, particularly under I/R conditions, and its inhibition may be, at least in part, responsible for COX inhibitor-induced AKI. Importantly, several studies have shown that the ability of the kidney to produce PGI_2_ decreases with aging [7, 33]. Whether reduced PGI_2_ production in aged people contributes to their increased susceptibility to AKI remains to be investigated.

For the first time, the present study shows in vivo that endothelial PGI_2_ plays a critical role in the cellular response to ischemic reperfusion and protects the kidney from injury using our floxed PGIS mice. By costaining PGIS with CD34, an endothelial marker, we show that PGIS is extensively expressed in the endothelial cells of the kidney. Our floxed PGIS mice allowed us to specifically explore the role of endothelial PGIS in renal I/R injury. We selectively deleted the PGIS gene in endothelial cells by crossing the floxed PGIS mice with an endothelial cell Cre mouse (TEK-CRE PGIS^fl/fl^). In an I/R-induced AKI model, the PGIS and PGI_2_ expression levels significantly increased after ischemia in the control kidneys but not in endothelial PGIS knockout kidneys. Selective deletion of endothelial PGIS markedly worsened the kidney damage following I/R, suggesting a protective role of endothelial PGI_2_ in the kidney. Deletion of PGIS did not alter the production of TXA_2_ and PGE_2,_ suggesting that the worsened renal damage was not caused by TXA_2_ or other prostanoids. Furthermore, administration of iloprost significantly attenuated the renal damage both in the control mice and endothelial PGIS knockout mice, supporting the protective role of PGI_2_. However, iloprost binds with equal affinity to IP and prostaglandin EP1 receptors and with lower affinity to EP3 receptors. Several studies show that high concentration of PGI_2_ can cause vasoconstriction under such conditions as NO or IP deficiency, and the vasoconstrictor effect of PGI_2_ is blocked by TP receptor antagonist [13, 26, 28]. Although these studies are important for us to understand the mechanism underlying hypertension or COX1-mediated vasoconstriction, whether the interaction of PGI_2_ and TP is involved in the protective effect of PGIS/PGI_2_ in AKI remains to be explored.

PGI_2_ has been reported to play an important role in modulating blood pressure by coupling with the IP receptor to relax vascular smooth muscle and control renin release [16, 22]. NSAIDs that inhibit COX and thus endogenous prostanoid synthesis may compromise the control of blood pressure in subjects with pre-existing salt-sensitive hypertension [14, 22]. In the present study, we examined the effect of PGIS deletion on systemic blood pressure. The result showed that the blood pressure of the TEK-CRE PGIS^fl/fl^ mice was not significantly different compared with wild-type mice, suggesting that the observed effect in the endothelial PGIS knockout was not mediated via its effect on blood pressure.

In our study, global deletion of the PGIS gene led to embryonic lethality, suggesting that the PGIS product is required for development. Mice with heterozygous PGIS deletion or with selective endothelial PGIS deletion were fertile and developed normally. No ischemic renal disorders were observed in heterozygous PGI_2_-deficient mice or endothelial PGI_2_-deficient TEK-CRE PGIS^fl/fl^ mice. Therefore, the observed effect of PGI_2_ deficiency on the kidney following I/R injury does not seem to be caused by abnormal renal development, although subtle developmental abnormality that may cause abnormal response to injury can not be excluded.

The protective effect of PGI_2_ against cellular stress has been reported [17, 23, 44]. However, the mechanism underlying its protective effect is incompletely understood and may vary according to the injury stresses. Several mechanisms through which PGIS and PGI_2_ exert their protective effects have been proposed, including anti-inflammatory mechanisms [9, 30, 38, 46], reducing oxidative stress [11, 41], and regulating renal blood flow [45]. It has been reported that prostacyclin and PPAR protect against I/RI by inducing an anti-inflammatory pathway [6]. Although we can show that PGIS deletion is associated with worsened kidney injury in an I/R model, PGIS deletion did not worsen the kidney injury in folic acid-induced AKI, which is a nephrotoxic model. Then, we used fluorescent microspheres to assess the effect of PGI_2_ on renal blood flow. Deletion of the PGIS gene in endothelial cells significantly reduced fluorescent microsphere accumulation in kidneys after reperfusion. A recent study showed that the restoration of microvascular perfusion is associated with reduced kidney injury [8]. However, this result is inconsistent with published results suggesting that the beneficial effect of PGI_2_ in renal I/R injury is not related to changes in renal blood flow [15]. The difference between these results may be due to how PGI_2_ was administered. Continuous infusion may cause hypotension and stimulate renin release, which would then block the reperfusion.

In summary, PGI_2_ expression was increased after I/R-induced AKI. The PGIS and PGI_2_ deficits in mouse kidneys increased vulnerability to I/R-induced AKI. Iloprost, an analog of PGI_2_, rescued the kidney from I/R-induced injury. This study provides evidence suggesting that PGIS-derived PGI_2_ can protect the kidney from acute injury caused by ischemic reperfusion and could be a potential preventive intervention for AKI.
